# Follistatin is a metastasis suppressor in a mouse model of HER2-positive breast cancer

**DOI:** 10.1186/s13058-017-0857-y

**Published:** 2017-06-05

**Authors:** Darcie D. Seachrist, Steven T. Sizemore, Emhonta Johnson, Fadi W. Abdul-Karim, Kristen L. Weber Bonk, Ruth A. Keri

**Affiliations:** 1 Department of Pharmacology, Case Western Reserve University School of Medicine, 10900 Euclid Avenue, Cleveland, OH 44106-4965 USA; 2 Department of Anatomic Pathology, Cleveland Clinic, Cleveland, OH 44195 USA; 3 Department of Genetics and Genome Sciences, Case Western Reserve University School of Medicine, Cleveland, OH 44106-4965 USA; 4 Division of General Medical Sciences-Oncology, Case Western Reserve University School of Medicine, Cleveland, OH 44106-4965 USA; 5 Present address: Department of Radiation Oncology, The James Comprehensive Cancer Center, The Ohio State University, Columbus, OH 43210 USA; 6 Present address: Department of Biology, Cuyahoga Community College, Cleveland, OH 44115 USA

**Keywords:** Breast cancer, Follistatin, Activin, Metastasis, Migration

## Abstract

**Background:**

Follistatin (FST) is an intrinsic inhibitor of activin, a member of the transforming growth factor-β superfamily of ligands. The prognostic value of FST and its family members, the follistatin-like (FSTL) proteins, have been studied in various cancers. However, these studies, as well as limited functional analyses of the FSTL proteins, have yielded conflicting results on the role of these proteins in disease progression. Furthermore, very few have been focused on FST itself. We assessed whether FST may be a suppressor of tumorigenesis and/or metastatic progression in breast cancer.

**Methods:**

Using publicly available gene expression data, we examined the expression patterns of *FST* and *INHBA*, a subunit of activin, in normal and cancerous breast tissue and the prognostic value of *FST* in breast cancer metastases, recurrence-free survival, and overall survival. The functional effects of activin and FST on in vitro proliferation, migration, and invasion of breast cancer cells were also examined. FST overexpression in an autochthonous mouse model of breast cancer was then used to assess the in vivo impact of FST on metastatic progression.

**Results:**

Examination of multiple breast cancer datasets revealed that *FST* expression is reduced in breast cancers compared with normal tissue and that low *FST* expression predicts increased metastasis and reduced overall survival. *FST* expression was also reduced in a mouse model of HER2/Neu-induced metastatic breast cancer. We found that FST blocks activin-induced breast epithelial cell migration in vitro, suggesting that its loss may promote breast cancer aggressiveness. To directly determine if FST restoration could inhibit metastatic progression, we transgenically expressed FST in the HER2/Neu model. Although FST had no impact on tumor initiation or growth, it completely blocked the formation of lung metastases.

**Conclusions:**

These data indicate that FST is a bona fide metastasis suppressor in this mouse model and support future efforts to develop an FST mimetic to suppress metastatic progression.

**Electronic supplementary material:**

The online version of this article (doi:10.1186/s13058-017-0857-y) contains supplementary material, which is available to authorized users.

## Background

Preventing metastatic progression is essential for extending the survival of patients with cancer, but the factors that control this process are not well understood. Indeed, there are relatively few proteins that have been identified as true metastasis suppressors (i.e., factors that block metastatic progression without impacting primary tumor growth). Such suppressors may function to inhibit intrinsic enhancers of metastatic progression. Activin, a member of the transforming growth factor-β (TGF-β) family, is a promigratory factor that is expressed in wounded tissues and promotes keratinocyte migration into the injured site [1]. Activin can also increase the in vitro migratory and invasive capacity of several epithelial cancer cell lines [2, 3]. These results suggest that activin may promote metastatic progression of cancer, but this supposition has not been fully assessed. This is in part due to the complexity of the activin protein family, which shares subunits with another class of proteins known as *inhibins*, as well as a lack of specificity of small-molecule inhibitors of the activin receptor. Supporting a prometastatic role for activin in breast cancer, elevated serum activin can predict the presence of bone metastases in patients [4]. Interconversion of differentiated breast cancer cells into stem cells also requires activin/nodal signaling [5], and one of the most predictive genes within a core metastasis-associated expression signature of multiple cancers is *INHBA*, which encodes an activin and inhibin subunit [6].

An endogenous and selective mechanism for inhibiting activin signaling is secretion of follistatin (FST), a protein that binds activin dimers with high affinity [7, 8]. Many tissues express both FST and activin, allowing FST to finely tune activin signaling [9]. Given the ability of FST to block activin signaling, it should prevent any prometastatic activities of activin. However, published studies suggesting a role for FST in cancer progression have been focused primarily on its prognostic value, and conflicting results have been reported [10]. Elevated serum FST correlates with the presence of bone metastases and increased PSA levels in patients with prostate cancer and has been proposed to be a therapeutic target in this disease [11, 12]. Similarly, increased FST expression is prognostic for poor overall survival in patients with ovarian, liver, and lung cancer [10]. In contrast, overexpression of FST in non-small cell lung cancer cells suppressed lung colonization following tail vein injection in immunocompromised mice [13]. The role of FST specifically in breast cancer progression is equally unclear. On the basis of immunohistochemistry (IHC), FST expression did not correlate with disease stage or recurrence in small cohorts of patients with breast cancer [14, 15]. Overexpression of FST inhibited growth of subcutaneous mammary carcinoma xenografts, but the impact of FST expression on metastatic progression has not yet been assessed [16]. The prognostic value of members of the FST family, the follistatin-like (FSTL) proteins, has also been studied, but, like FST, their role in tumorigenesis and metastasis is unresolved [17–20].

By examining intrinsic changes that occur in a mouse model of HER2/Neu (human epidermal growth factor receptor 2, receptor tyrosine protein kinase erbB-2, proto-oncogene *Neu*)-induced metastatic breast cancer, we previously reported that activin receptor-like kinase 4 and phosphorylated Smad2 are present within tumors, whereas tumors had lost expression of transforming growth factor β-receptor 1 (TGF-βR1) [21]. Hence, Smad2 signaling could be activated in mammary cancers in the absence of TGF-β signaling, and this was likely due to the presence of activin. Given the potential impact of activin on cancer progression, as well as the ability of FST to block the binding of activin to its receptor, we postulated that FST may act as a metastasis suppressor. In this study, we show that expression of the activin subunit *INHBA* is elevated in multiple cohorts of breast cancers, whereas *FST* is suppressed. We also report that FST suppresses activin-induced migration of human breast cancer cells. Most important, enforced expression of FST in the mouse mammary gland completely blocks metastases in a model of HER2/Neu-positive breast cancer without having any impact on primary tumor latency or growth. These studies reveal that FST is an intrinsic inhibitor of metastatic progression.

## Methods

### Datasets

Gene expression data were retrieved from the online Oncomine platform (http://www.oncomine.com/). Statistical comparisons of *INHBA* and *FST* expression in normal breast versus breast cancer in The Cancer Genome Atlas [22] dataset or in normal breast stroma versus breast cancer-associated stroma in the Finak et al. dataset [23] were performed by Student’s *t* test. For statistical analysis of the prognostic value of *FST* expression in recurrence to bone or brain/lung in the Bos et al. dataset [24], the respective patient cohort was stratified into high (upper 10th percentile) and low (remaining 90th percentile) groups. A detailed description of the Kaplan-Meier analyses is provided in Additional file 1.

### Transgenic mice

FST-overexpressing mice were generated as previously described [25], and founders were bred with FVB/N males (The Jackson Laboratory, Bar Harbor, ME, USA) to expand the lines. Neu/FST bitransgenic mice were obtained by crossing FST-overexpressing females with homozygous FVB-transgenic (Tg) (mouse mammary tumor virus [MMTV]-Erbb2) NK1Mul/J [26] males (HER2/Neu) obtained from The Jackson Laboratory. Age-matched littermates were used for comparisons in all studies. Mice were palpated weekly for tumor formation, and, once detected, tumors were measured with calipers. Tumor volumes were calculated as previously described [21]. Mice were housed in microisolator cages under pathogen-free conditions with a 12-h/12-h light/dark cycle and were provided food and water ad libitum. All animal studies were approved by the Case Western Reserve University Institutional Animal Care and Use Committee.

### Cell culture

MCF10A, MCF7, T47D, BT474, SKBR3, MDA-MB-231, and MDA-MB-468 cells were obtained from the American Type Culture Collection (ATCC, Manassas, VA, USA) and lines that were maintained for more than three years were authenticated by short tandem repeat profiling (Barbara Davis Center Molecular Biology Service Center, University of Colorado, Boulder, CO, USA). MCF10A-Neu cells were previously described [27]. Cells were cultured according to ATCC instructions and maintained in 5% CO_2_ at 37 °C in a humidified incubator.

### Histology and immunohistochemistry

Tissues were fixed in 4% paraformaldehyde and processed as previously described [21]. For FST IHC, goat α-FST (AF669; R&D Systems, Minneapolis, MN, USA), or immunoglobulin G as a negative control, was applied at 2 μg/ml overnight at 4 °C. To determine the relative number of metastases/emboli present in the lungs per mouse, sections of lung were collected every 100 μm and stained with hematoxylin and eosin. The number of metastases or emboli per section was quantified by a blinded pathologist.

### Western blotting

Cells were lysed in radioimmunoprecipitation assay buffer, diluted in Laemmli buffer, run on a 10% SDS-PAGE gel, and transferred onto a polyvinylidene difluoride membrane. Blots were incubated in the appropriate secondary antibody tagged with HRP. Bound antibody was detected by chemiluminescence (SignalFire ECL reagent; Cell Signaling Technology, Danvers, MA, USA).

### RNA analyses

RNA was isolated using Invitrogen TRIzol reagent (Life Technologies, Carlsbad, CA, USA) and DNase (Ambion DNA-free kit; Life Technologies). Complementary DNA was generated using SuperScript II reverse transcriptase and random hexamers (Life Technologies) as per the manufacturer’s protocol. Quantitative real-time polymerase chain reaction (qPCR) was performed on a StepOnePlus Real-Time PCR system using TaqMan gene expression assays (Applied Biosystems, Foster City, CA, USA) for mouse *Fst* (Mm00514982_m1) or human *FST* (*Hs00246256_m1*) and normalized to *Gapdh* (4352932-0804021) or *TBP* (*Hs00427620_m1*), respectively.

### Migration and invasion assays

Cells were plated and treated with vehicle, and then treated with previously reported doses of recombinant human activin A (100 ng/ml; R&D Systems) and/or recombinant human FST (400 ng/ml; R&D Systems) for 48 h in serum-free media [28–30]. Cells were plated for migration in modified Boyden chambers (Costar Transwell Permeable Supports, catalogue number 3422, Corning Life Sciences, Tewksbury, MA, USA) or invasion in Transwell inserts coated with Matrigel (Corning BioCoat Matrigel Invasion Chamber, catalogue number 354480; Corning Life Sciences). The upper chamber contained the treatment in serum-free media, and the bottom chamber contained treatment in complete media. Cells were allowed to migrate for 16–24 h. Filters were stained with Diff-Quick. The number of migrated cells per filter was calculated by averaging the number of cells per × 10 magnification field in five independent fields. Three independent experiments were performed in duplicate.

### Proliferation/viability assays

Cells were plated and treated with vehicle, recombinant human activin A (100 ng/ml; R&D Systems), and/or recombinant human FST (400 ng/ml; R&D Systems) for 72 h in serum-free media. Cell number was quantified using the CellTiter 96 AQueous One Solution Cell Proliferation Assay kit (Promega, Madison, WI, USA) according to the manufacturer’s instructions.

### Statistical analyses

Data are expressed as the mean ± SD. Statistical analyses were performed using one-way analysis of variance followed by Student’s *t* test, a log-rank test, or Fisher’s exact test where indicated. A minimum *p* value <0.05 was considered statistically significant.

## Results

**Fig. 1:**
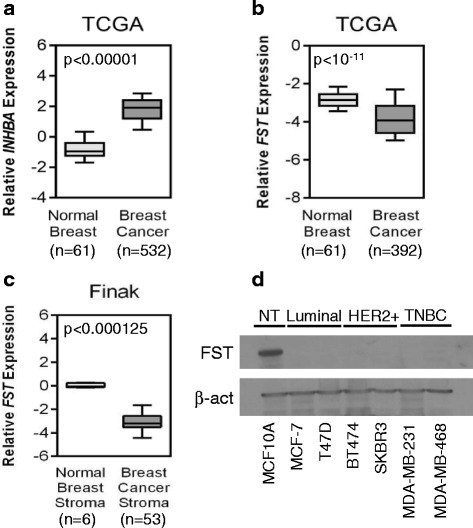
Expression of the INHBA/follistatin (FST) axis is altered in human breast cancers. Relative (**a**) *INHBA* and (**b**) *FST* expression in normal breast versus breast cancer using publicly available datasets from The Cancer Genome Atlas (TCGA). **c** Relative *FST* expression in normal breast stroma versus breast cancer stroma in the Finak et al*.* dataset. **d** Western blot analysis of FST protein expression in immortalized, nontransformed mammary epithelial cells (NT) and breast cancer cell lines representing luminal, human epidermal growth factor receptor 2-positive (HER2+), and triple-negative breast cancer (TNBC) subtypes

To determine if activin may have a role in breast cancer, we examined several publicly available gene expression datasets and found that the gene encoding the activin A subunit, *INHBA*, is upregulated in the majority of breast tumors compared with normal breast tissue in three independent datasets (Fig. 1a and Additional file 2: Figure S1a). Moreover, expression of *FST*, the endogenous inhibitor of activin, was downregulated in breast cancer tissue as well as surrounding stroma compared with normal breast tissue (Fig. 1b and c and Additional file 2: Figure S1b). Together, these data suggest that maximal activin signaling occurs in the breast tumor microenvironment. The suppression of *FST* was mirrored in breast cancer cell lines compared with immortalized, nontransformed human mammary epithelial cell lines (MCF10A and MCF12A) and was independent of molecular subtype or receptor status (Fig. 1d and Additional file 3: Figure S2a), suggesting that suppression of *FST* expression may be an integral step in breast tumorigenesis, regardless of the oncogenic driver. Supporting this possibility, overexpression of the proto-oncogene HER2/c-Neu in nontransformed MCF10A cells [27] also suppressed *FST* expression (Additional file 3: Figure S2b), indicating that the nascent activation of an oncogenic pathway is sufficient to suppress *FST* expression.

**Fig. 2:**
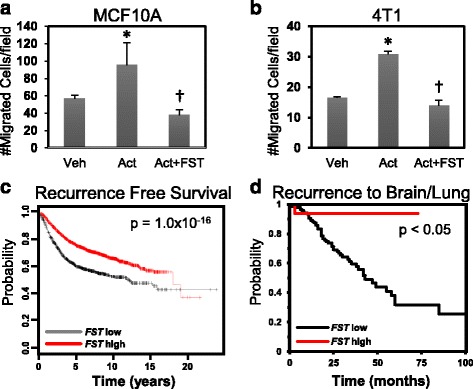
Follistatin (FST) treatment inhibits migration, and elevated *FST* expression is correlated with better breast cancer outcomes. Modified Boyden chamber migration assays of (**a**) MCF10A and (**b**) 4T1 cells treated with vehicle (Veh), 100 ng/ml recombinant human (rh) activin A (Act), or activin A plus rhFST (400 ng/ml). Migrated cells were quantified in five independent fields per filter and normalized to vehicle (*p < 0.05 compared with vehicle, ^†^ *p* < 0.05 compared with activin). **c** Kaplan-Meier analysis of recurrence-free survival of patients with breast cancer with tumors stratified by *FST* expression using a 3554-patient cohort representing all breast cancer subtypes (*FST* low, *n* = 967; *FST* high, *n* = 2587). **d** Kaplan-Meier analysis of the prognostic significance of *FST* expression on breast cancer metastasis to the brain/lung in the Bos et al*.* [24] cohort (FST low, *n* = 149; FST high, *n* = 17)

To assess the impact of FST on activin-induced migration of breast epithelial cells, we treated MCF10A and 4T1 cells (a mouse mammary cancer cell line) with activin A in the presence or absence of recombinant FST. Activin stimulated the migration of these cells, and FST suppressed this induction (Fig. 2a and b), without altering cell number (Additional file 4: Figure S3a). Previous studies have shown reduced proliferation of breast cancer cell lines upon activin treatment or FSTL3 silencing in vitro. However, estrogen receptor-expressing cell lines were used in these studies [28, 31, 32], and it has been reported that activin and estrogen have opposing effects on proliferation [33]. Thus, the ability of activin to modulate proliferation of mammary epithelial cells may be context-dependent. In addition to migration, FST also suppressed activin-induced invasion of MCF10A cells overexpressing HER2/Neu (Additional file 4: Figure S3b), further indicating that FST suppression may be necessary during metastatic progression. Supporting this possibility, a meta-analysis of 2878 patients with breast cancer (all subtypes) revealed that sustained *FST* messenger RNA (mRNA) expression correlates with prolonged recurrence-free survival compared with patients whose tumors had low *FST* expression (Fig. 2c). The impact on survival was similar to that observed with increased estrogen receptor-1 (*ESR1*) expression, a marker of more differentiated cancers (Additional file 5: Figure S4). *FST* expression also correlates with increased overall survival and reduced metastasis in four independent datasets and is particularly prognostic of reduced recurrence in brain and lung but not in bone (Fig. 2d and Additional file 6: Figure S5). The association between *FST* and recurrence was independent of breast cancer subtype (Additional file 7: Figure S6), suggesting that it may be a global repressor of breast cancer progression. Of note, genes encoding the related proteins, FSTL1, FSTL2, and FSTL3, which can also bind and inhibit activin signaling, were also prognostic of recurrence-free survival, although *FSTL2* had very modest prognostic ability (Additional file 8: Figure S7).

**Fig. 3:**
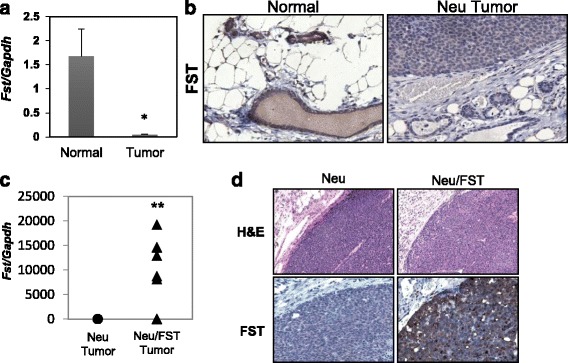
Follistatin (FST) expression is repressed in mouse mammary tumors and is restored using an *Fst*-expressing transgene. **a** Endogenous *Fst* expression in normal mouse mammary glands compared with human epidermal growth factor receptor 2, receptor tyrosine protein kinase erbB-2, proto-oncogene *Neu* (Her2/Neu)-induced mammary tumors (*p < 0.05). **b** Immunohistochemical analysis of endogenous FST expression in mouse mammary epithelia and tumors. **c** *Fst* expression in Neu (single) and Neu/FST (bitransgenic) tumors. *Fst* messenger RNA expression was assessed by quantitative reverse transcription-polymerase chain reaction (**p < 0.01). **d** Hematoxylin and eosin (H&E)-stained mammary tumors from Neu (single) and Neu/FST (bitransgenic) mice are shown in the *upper two panels*. Immunohistochemistry for total (endogenous and transgenic) FST expression in *lower two panels* in single (*left*) and bitransgenic (*right*) tumors

**Fig. 4:**
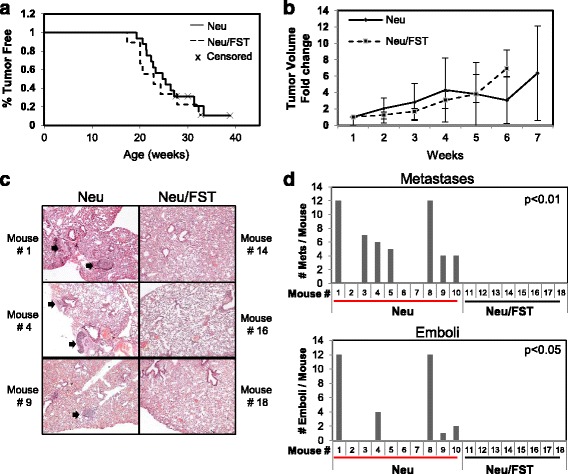
Restoring follistatin (FST) expression abrogates lung metastases in a mouse model of metastatic breast cancer. **a** Kaplan-Meier plot comparing the percentage of tumor-free mice in a cohort of single transgenic Neu (*n* = 11) and bitransgenic Neu/FST (*n* = 8) mice. **b** Fold change in tumor volume after detection by palpation in single and bitransgenic cohorts. **c** Representative × 4 magnification images of hematoxylin and eosin-stained lung sections depicting metastases in single and bitransgenic mice. *Arrows* indicate metastases. **d** Quantitation of the number of true lung metastases (*p* < 0.01) and emboli (*p* < 0.05) in each mouse examined (Neu, *n* = 10 mice; Neu/FST, *n* = 8 mice). Statistical significance was determined using Fisher’s exact test

As in human breast cancer, we found that FST was profoundly repressed in mammary tumors collected from transgenic mice expressing the Her2/Neu proto-oncogene in the mammary epithelia (MMTV-Neu) (Fig. 3a and b), as was *Fstl3* (Additional file 9: Figure S8a) [34]. Thus, mice expressing Her2/Neu in the mammary gland provide an ideal model to determine if FST loss is necessary for metastatic progression of cancer by restoring expression of FST in mammary tumors. We generated transgenic mice that selectively overexpress FST in the mammary epithelium using the MMTV-long terminal repeat promoter [25, 35]. Founder female mice and subsequent female offspring were viable and fertile. They also displayed a greater than 200-fold elevation in *Fst* expression in their mammary glands (Additional file 9: Figure S8b and c), but no overt differences were detected in mammary ductal outgrowth of female MMTV-Fst progeny (Additional file 9: Figure S8e). *Fst* expression was also modestly elevated in the uterus and lungs of adult transgenic females compared with wild-type female littermates, with no increase of *Fst* occurring in the liver (Additional file 9: Figure S8d). FST-overexpressing mice were then crossed with MMTV-Neu (HER 2/Neu) mice that express a constitutively active form of the HER 2/Neu receptor [26] to generate single (Neu) and bitransgenic (Neu/FST) littermates (Additional file 9: Figure S8f). The resultant females were palpated weekly for tumor formation, and FST overexpression in the tumor epithelium was confirmed by qPCR and IHC (Fig. 3c and d). Restoring FST expression in HER 2/Neu tumors had no impact on tumor latency or tumor volume (Fig. 4a and b, respectively). Neu/FST bitransgenic tumors also showed no overt differences in morphology compared with single transgenic Neu tumors, despite abundant FST protein expression throughout the tumor epithelium (Fig. 3d). In contrast to the lack of effect of the FST transgene on tumor initiation, a major impact on metastatic progression was observed. Whereas 72% and 40% of Her2/Neu single transgenic littermates displayed multiple lung metastases and lymphovascular emboli, respectively, no metastases were evident in Neu/FST bitransgenic mouse lungs (Fig. 4c and d). These data indicate that FST is a potent and bona fide metastasis suppressor in this model of breast cancer.

## Discussion

This is the first report demonstrating that *FST* expression is reduced in breast cancers compared with normal breast tissue and that elevated *FST* mRNA is associated with delayed recurrence and survival in patients with breast cancer. We evaluated *FST* expression in breast cancer versus normal breast in three independent breast cancer datasets totaling more than 2000 breast cancer samples and over 200 samples of normal breast. Previous analyses of FST expression at the protein level have not demonstrated differential expression between normal breast and breast cancer in small patient cohorts [15, 32]. The researchers in these studies evaluated a small population of tumors (*n* = 22 invasive cancers in the largest cohort) and used tumor-adjacent tissue as the “normal breast” comparator. Several studies have shown that tumor-adjacent tissue is poised for malignancy with differential expression patterns being observed between normal tissue and cancer in both human and mouse [21, 36, 37]. This may be particularly true when evaluating secreted factors such as activin and FST that have pleiotropic effects on tissues. It is also possible that there is discordance between FST mRNA and protein expression in breast cancers. To address this, large-scale analyses using more quantitative measures such as reverse phase protein array or western blotting combined with outcome data are necessary to determine if FST protein expression in tumors is an accurate prognosticator of recurrence and survival.

Data reported herein directly show that sustained expression of FST prevents metastatic progression in a mouse model of breast cancer. This adds *FST* to the short list of metastasis suppressor genes, such as breast cancer metastasis suppressor-1 (*BRMS1*), cadherin-1 (*CDH1*), and nucleoside diphosphate kinase 1 (*NM23*), that inhibit metastasis formation without impacting primary tumor growth [38]. Whereas we found that FST expression had no impact on tumor growth in the MMTV-Neu model, a previous report indicated that ectopic expression of FST decreased growth of R30C breast cancer xenografts compared with those overexpressing activin [16]. These conflicting results likely reflect the use of divergent model systems. The R30C study involved the subcutaneous injection of a modestly characterized breast cancer cell line (R30C) into severe combined immunodeficiency mice. These cells lack an estrogen receptor, and it is unknown if they express HER2/Neu [39]. In contrast, we used a credentialed and autochthonous model of HER2/Neu-positive breast cancer in which the FST transgene was expressed during the transformation/progression process and likely impacted the mammary microenvironment. Furthermore, the transgenic model used in our studies has an intact immune system. It is well established that the immunoreactivity can enhance tumor growth and metastatic disease [40, 41], and we expect that any immunomodulatory impact of FST/activin should be observed in this model. Thus, the differences observed when examining the impact of FST on tumor growth may be reflective of the different approaches used. More important, in our study, we specifically examined the impact of FST expression on metastatic progression, whereas Krneta et al., focused on the role of FST and activin in primary tumor angiogenesis and growth without a specific examination of metastatic impact.

The ability of FST to prevent metastatic progression, combined with the propensity for FST to bind and inhibit activin signaling, suggests that activin secreted from the microenvironment may be a key driver of metastatic progression in breast cancer, similarly to TGF-β. In support of this possibility, the activin receptor colocalizes with phosphorylated Smad2 at the leading edge of HER 2/Neu mouse tumors [21]. However, FST can also bind and inhibit myostatin, growth/differentiation factor-9 (GDF-9), and several bone morphogenetic protein (BMP) family members, albeit with lower affinity [42, 43]. Although there is little evidence that myostatin or GDF-9 is expressed in breast cancer, inhibition of the BMP receptor, activin receptor-like kinase 2 (ALK2), can reduce metastatic spread of mammary tumors in mice. Treatment of MMTV-polyoma virus middle T antigen mice with the selective ALK2 antagonist DMH1 reduced lung metastases but did not eliminate them altogether [44]. This contrasts with FST expression, where we observed a complete loss of metastases in the HER2/Neu model. Furthermore, DMH1-treated tumors displayed decreased fibrosis, lymphatic vessels, and macrophage infiltration, whereas expression of FST in the MMTV-Neu model did not alter the histomorphology of tumors, including the extent of desmoplasia, macrophage infiltration, or vascular density (data not shown), suggesting distinct mechanisms of action. Moreover, low BMP mRNA levels predict poorer breast cancer prognoses (data not shown), which conflicts with the proposed prometastatic role of BMPs in the mouse model. In contrast to BMPs, elevated expression of the activin subunit *INHBA* predicts worse outcomes in patients with breast cancer and is consistent with our observation that FST prevents metastasis. As a whole, these data reveal that FST suppresses metastatic progression and suggest that this effect is due primarily to suppression of activin rather than to major effects on BMP signaling.

Several studies suggest that elevated serum activin is associated with tumor progression and prognosis. However, the potential for using serum activin as a marker of breast cancer metastasis is not clear. Elevated serum activin is found in patients with breast cancer with bone metastases, but it is not associated with metastasis to the lymph nodes [45, 46]. Since activin was first isolated from ovarian follicular fluid, the number of tissues that have been reported to produce activin has grown exponentially, complicating its use as a serum biomarker [47]. Indeed, elevated activin is found not only in the serum of patients with cancer but also in patients with septicemia, Graves’ disease, and heart failure [48–50], suggesting that activin may be an indicator of systemic inflammation. This is unsurprising because activin plays a role in the wound response, and immune cells produce high amounts of activin [51, 52]. On the basis of our previous observation that activin receptor colocalizes with phosphorylated Smad2 at the tumor-stroma interface, we postulate that activin from the tumor microenvironment promotes metastatic progression of breast cancer and that restoration of FST is sufficient to inhibit this process [21, 45, 46].

## Conclusions

The data we report demonstrate that FST is a potent and bona fide metastasis suppressor in a model of breast cancer and suggest that approaches aimed at restoring FST levels, mimicking FST activity, or blocking its target, activin, should be examined as a potential avenue for extending metastasis-free survival of patients with breast cancer.

## Additional files

Additional file 1: Supplementary methods. (DOCX 21 kb)

Additional file 2: Figure S1. Expression of the INHBA/FST axis is altered in human breast cancers. **a** Relative *INHBA* expression in normal breast versus breast cancer using publicly available datasets from Richardson et al. [56] and Sorlie et al. [57]. **b** Relative *FST* expression in normal breast versus breast cancer as above from Richardson et al. [56] and Curtis et al. [54] datasets. (PPTX 161 kb)

Additional file 3: Figure S2. FST expression is reduced in breast cancer cell lines compared with nontransformed mammary epithelial cells. **a** Western blot analysis of breast cancer cell lines demonstrating loss of *FST* expression compared with nontransformed (NT) mammary epithelial cells. **b** *FST* expression in MCF10A versus MCF10A-Neu stable cell lines that overexpress rat c-Neu/ErbB2 [27]. *FST* was assessed by quantitative RT-PCR relative to TATA-binding protein (*TBP*) mRNA (**p < 0.01). (PPTX 1925 kb)

Additional file 4: Figure S3. Follistatin inhibits activin A-induced invasion without impacting proliferation. **a** MCF10A and 4 T1 cells were treated with vehicle, recombinant human activin A (100 ng/ml), or activin A plus recombinant human FST (400 ng/ml), and cell number was assessed by MTS assay after 72 h. **b** MCF10A cells that overexpress rat c-Neu (10ANeu) were treated with vehicle, activin A (100 ng/ml), or activin A plus FST (400 ng/ml) for 48 h and plated for invasion assays in modified Boyden chambers + Matrigel overnight with serum as a chemoattractant in addition to follistatin and/or activin A (*p < 0.01 compared with vehicle and ^†^ *p* < 0.01 compared with activin A). (PPTX 115 kb)

Additional file 5: Figure S4. Kaplan-Meier plot demonstrating that *ESR1* expression predicts recurrence-free survival similarly to *FST* in a cohort of over 2800 patients with breast cancer (all subtypes). Patients are stratified in high- and low-expressing groups for *ESR1* using optimal cutoffs in the KM Plotter data analysis tool [58]. (PPTX 84 kb)

Additional file 6: Figure S5. FST expression is associated with overall survival and metastasis in multiple cohorts of patients with breast cancer. Tumors are stratified into the highest 10% and lowest (remaining 90%) *FST-*expressing groups for each dataset as follows: Curtis et al. [54] reported FST low (*n* = 1774), FST high (*n* = 197); Hatzis et al. [53] reported FST low (*n* = 457), FST high (*n* = 51); and Kao et al. [55] reported FST low (*n* = 294), FST high (*n* = 33)*.* FST expression does not predict recurrence to bone in the Bos et al. cohort [24]: FST low (*n* = 149), FST high (*n* = 17). (PPTX 109 kb)

Additional file 7: Figure S6. FST expression predicts recurrence-free survival independent of breast cancer subtype. Kaplan-Meier plots demonstrating the association of high *FST* expression with recurrence-free survival of luminal A, luminal B, HER2, and basal subtypes of breast cancer. High and low *FST*-expressing groups are stratified using optimal cutoffs in the KM Plotter data analysis tool [58]. (PPTX 330 kb)

Additional file 8: Figure S7. Kaplan-Meier plots demonstrating that FSTL1, FSTL2, and FSTL3 expression also predict recurrence-free survival in a cohort of over 2800 patients with breast cancer. Patients are stratified in high- and low-expressing groups for each gene using optimal cutoffs in the KM Plotter data analysis tool [58]. (PPTX 185 kb)

Additional file 9: Figure S8. FST overexpression in mouse mammary epithelia. **a** Follistatin-like 3 (*Fstl3*) is downregulated in HER 2/Neu-induced mouse mammary tumors compared with normal mammary glands. *Fstl3* expression in mammary glands and HER 2/Neu tumors was determined using data from a published microarray study of these tumors (*p < 0.001) [21]. **b** and **c** *Fst* is overexpressed in mammary epithelia of founder female mice and *Fst*-overexpressing founder offspring (in mixed FVB/N background) as determined by quantitative RT-PCR. **d** *Fst* expression in uterus, lung, and liver of 10- to 12-month-old MMTV-*Fst* female single transgenic mice (*n* = 3) and wild-type littermates (*n* = 2). **e** Representative whole mounts of adult mammary glands from female *Fst*-overexpressing founder offspring and littermate wild-type mice in the C57BL/6:SJ:FVB background. **f** Schematic of MMTV-*Fst* transgene and breeding paradigm used to obtain FST/Neu bitransgenic mice with restored FST expression. (PPTX 712 kb)

## Abbreviations

ALK2: Activin receptor-like kinase 2
ATCC: American Type Culture Collection
BMP: Bone morphogenetic protein
CDH1: Cadherin-1
ESR1: Estrogen receptor 1
FST: Follistatin
FSTL: Follistatin-like
GDF-9: Growth/differentiation factor 9
H&E: Hematoxylin and eosin
HER2/neu: Human epidermal growth factor receptor 2, receptor tyrosine protein kinase erbB-2, proto-oncogene *Neu*
IHC: Immunohistochemistry
MMTV: Mouse mammary tumor virus
mRNA: Messenger RNA
NM23: Nucleoside diphosphate kinase 1
NT: Nontransformed
qPCR: Quantitative real-time polymerase chain reaction
rh: Recombinant human
TCGA: The Cancer Genome Atlas
Tg: Transgenic
TGF-β: Transforming growth factor β
TGF-βR1: Transforming growth factor β-receptor 1
TNBC: Triple-negative breast cancer

## References

[1] Zhang L, Deng M, Parthasarathy R, Wang L, Mongan M, Molkentin JD (2005). MEKK1 transduces activin signals in keratinocytes to induce actin stress fiber formation and migration. Mol Cell Biol.

[2] Neel JC, Lebrun JJ (2013). Activin and TGFβ regulate expression of the microRNA-181 family to promote cell migration and invasion in breast cancer cells. Cell Signal.

[3] Taylor C, Loomans HA, Le Bras GF, Koumangoye RB, Romero-Morales AI, Quast LL (2015). Activin A signaling regulates cell invasion and proliferation in esophageal adenocarcinoma. Oncotarget.

[4] Leto G, Incorvaia L, Flandina C, Ancona C, Fulfaro F, Crescimanno M (2016). Clinical impact of cystatin C/cathepsin L and follistatin/activin A systems in breast cancer progression: a preliminary report. Cancer Invest.

[5] Meyer MJ, Fleming JM, Ali MA, Pesesky MW, Ginsburg E, Vonderhaar BK (2009). Dynamic regulation of CD24 and the invasive, CD44^pos^CD24^neg^ phenotype in breast cancer cell lines. Breast Cancer Res.

[6] Kim H, Watkinson J, Varadan V, Anastassiou D (2010). Multi-cancer computational analysis reveals invasion-associated variant of desmoplastic reaction involving INHBA, THBS2 and COL11A1. BMC Med Genomics.

[7] Phillips DJ, de Kretser DM (1998). Follistatin: a multifunctional regulatory protein. Front Neuroendocrinol.

[8] Sugino H, Sugino K, Hashimoto O, Shoji H, Nakamura T (1997). Follistatin and its role as an activin-binding protein. J Med Invest.

[9] Welt C, Sidis Y, Keutmann H, Schneyer A (2002). Activins, inhibins, and follistatins: from endocrinology to signaling. A paradigm for the new millennium. Exp Biol Med.

[10] Shi L, Resaul J, Owen S, Ye L, Jiang WG (2016). Clinical and therapeutic implications of follistatin in solid tumours. Cancer Genomics Proteomics.

[11] Tumminello FM, Badalamenti G, Fulfaro F, Incorvaia L, Crescimanno M, Flandina C (2010). Serum follistatin in patients with prostate cancer metastatic to the bone. Clin Exp Metastasis.

[12] Sepporta MV, Tumminello FM, Flandina C, Crescimanno M, Giammanco M, La Guardia M (2013). Follistatin as potential therapeutic target in prostate cancer. Target Oncol.

[13] Ogino H, Yano S, Kakiuchi S, Muguruma H, Ikuta K, Hanibuchi M (2008). Follistatin suppresses the production of experimental multiple-organ metastasis by small cell lung cancer cells in natural killer cell–depleted SCID mice. Clin Cancer Res.

[14] Couto HL, Dela Cruz C, Buzelin MA, Toppa NH, Wainstein AJ, Reis FM. Follistatin expression in human invasive breast tumors: pathologic and clinical associations. Appl Immunohistochem Mol Morphol. doi:10.1097/PAI.0000000000000385. 10.1097/PAI.0000000000000385 27389553

[15] Bloise E, Couto HL, Massai L, Ciarmela P, Mencarelli M, Borges LE (2009). Differential expression of follistatin and FLRG in human breast proliferative disorders. BMC Cancer.

[16] Krneta J, Kroll J, Alves F, Prahst C, Sananbenesi F, Dullin C (2006). Dissociation of angiogenesis and tumorigenesis in follistatin- and activin-expressing tumors. Cancer Res.

[17] Kudo-Saito C (2013). FSTL1 promotes bone metastasis by causing immune dysfunction. Oncoimmunology.

[18] Kudo-Saito C, Fuwa T, Murakami K, Kawakami Y (2013). Targeting FSTL1 prevents tumor bone metastasis and consequent immune dysfunction. Cancer Res.

[19] Zhao W, Han HB, Zhang ZQ (2011). Suppression of lung cancer cell invasion and metastasis by connexin43 involves the secretion of follistatin-like 1 mediated via histone acetylation. Int J Biochem Cell Biol.

[20] Couto HL, Buzelin MA, Toppa NH, Bloise E, Wainstein AJ, Reis FM. Prognostic value of follistatin-like 3 in human invasive breast cancer. Oncotarget. doi:10.18632/oncotarget.15026. 10.18632/oncotarget.15026 PMC5522059 28178680

[21] Landis MD, Seachrist DD, Montañez-Wiscovich ME, Danielpour D, Keri RA (2005). Gene expression profiling of cancer progression reveals intrinsic regulation of transforming growth factor-β signaling in ErbB2/Neu-induced tumors from transgenic mice. Oncogene.

[22] The Cancer Genome Atlas Network (2012). Comprehensive molecular portraits of human breast tumours. Nature.

[23] Finak G, Bertos N, Pepin F, Sadekova S, Souleimanova M, Zhao H (2008). Stromal gene expression predicts clinical outcome in breast cancer. Nat Med.

[24] Bos PD, Zhang XHF, Nadal C, Shu W, Gomis RR, Nguyen DX (2009). Genes that mediate breast cancer metastasis to the brain. Nature.

[25] Seachrist DD, Johnson E, Magee C, Clay CM, Graham JK, Veeramachaneni DN (2012). Overexpression of follistatin in the mouse epididymis disrupts fluid resorption and sperm transit in testicular excurrent ducts. Biol Reprod.

[26] Muller WJ, Sinn E, Pattengale PK, Wallace R, Leder P (1988). Single-step induction of mammary adenocarcinoma in transgenic mice bearing the activated c-*neu* oncogene. Cell.

[27] Johnson E, Seachrist DD, DeLeon-Rodriguez CM, Lozada KL, Miedler J, Abdul-Karim FW (2010). HER2/ErbB2-induced breast cancer cell migration and invasion require p120 catenin activation of Rac1 and Cdc42. J Biol Chem.

[28] Burdette JE, Jeruss JS, Kurley SJ, Lee EJ, Woodruff TK (2005). Activin A mediates growth inhibition and cell cycle arrest through Smads in human breast cancer cells. Cancer Res.

[29] Salogni L, Musso T, Bosisio D, Mirolo M, Jala VR, Haribabu B (2009). Activin A induces dendritic cell migration through the polarized release of CXC chemokine ligands 12 and 14. Blood.

[30] Kipp JL, Kilen SM, Woodruff TK, Mayo KE (2007). Activin regulates estrogen receptor gene expression in the mouse ovary. J Biol Chem.

[31] Liu QY, Niranjan B, Gomes P, Gomm JJ, Davies D, Coombes RC (1996). Inhibitory effects of activin on the growth and morphogenesis of primary and transformed mammary epithelial cells. Cancer Res.

[32] Razanajaona D, Joguet S, Ay AS, Treilleux I, Goddard-Leon S, Bartholin L (2007). Silencing of FLRG, an antagonist of activin, inhibits human breast tumor cell growth. Cancer Res.

[33] Burdette JE, Woodruff TK (2007). Activin and estrogen crosstalk regulates transcription in human breast cancer cells. Endocr Relat Cancer.

[34] Guy CT, Webster MA, Schaller M, Parsons TJ, Cardiff RD, Muller WJ (1992). Expression of the *neu* protooncogene in the mammary epithelium of transgenic mice induces metastatic disease. Proc Natl Acad Sci U S A.

[35] Wagner KU, Wall RJ, St-Onge L, Gruss P, Wynshaw-Boris A, Garrett L (1997). Cre-mediated gene deletion in the mammary gland. Nucleic Acids Res.

[36] Chandran UR, Dhir R, Ma C, Michalopoulos G, Becich M, Gilbertson J (2005). Differences in gene expression in prostate cancer, normal appearing prostate tissue adjacent to cancer and prostate tissue from cancer free organ donors. BMC Cancer.

[37] Tian F, Li R, Chen Z, Shen Y, Lu J, Xie X (2016). Differentially expressed miRNAs in tumor, adjacent, and normal tissues of lung adenocarcinoma. Biomed Res Int.

[38] Yan J, Yang Q, Huang Q (2013). Metastasis suppressor genes. Histol Histopathol.

[39] Bargou RC, Wagener C, Bommert K, Mapara MY, Daniel PT, Arnold W (1996). Overexpression of the death-promoting gene bax-α which is downregulated in breast cancer restores sensitivity to different apoptotic stimuli and reduces tumor growth in SCID mice. J Clin Invest.

[40] Kitamura T, Qian BZ, Pollard JW (2015). Immune cell promotion of metastasis. Nat Rev Immunol.

[41] Loomans HA, Andl CD (2015). Intertwining of activin A and TGFβ signaling: dual roles in cancer progression and cancer cell invasion. Cancers.

[42] Schneyer AL, Sidis Y, Gulati A, Sun JL, Keutmann H, Krasney PA (2008). Differential antagonism of activin, myostatin and growth and differentiation factor 11 by wild-type and mutant follistatin. Endocrinology.

[43] Pentek J, Parker L, Wu A, Arora K (2009). Follistatin preferentially antagonizes activin rather than BMP signaling in *Drosophila*. Genesis.

[44] Owens P, Pickup MW, Novitskiy SV, Giltnane JM, Gorska AE, Hopkins CR (2015). Inhibition of BMP signaling suppresses metastasis in mammary cancer. Oncogene.

[45] Leto G, Incorvaia L, Badalamenti G, Tumminello FM, Gebbia N, Flandina C (2006). Activin A circulating levels in patients with bone metastasis from breast or prostate cancer. Clin Exp Metastasis.

[46] Reis FM, Cobellis L, Tameirao LC, Anania G, Luisi S, Silva IS (2002). Serum and tissue expression of activin a in postmenopausal women with breast cancer. J Clin Endocrinol Metab.

[47] Ling N, Ying SY, Ueno N, Shimasaki S, Esch F, Hotta M (1986). Pituitary FSH is released by a heterodimer of the β-subunits from the two forms of inhibin. Nature.

[48] Yndestad A, Ueland T, Øie E, Florholmen G, Halvorsen B, Attramadal H (2004). Elevated levels of activin A in heart failure: potential role in myocardial remodeling. Circulation.

[49] Michel U, Ebert S, Phillips D, Nau R (2003). Serum concentrations of activin and follistatin are elevated and run in parallel in patients with septicemia. Eur J Endocrinol.

[50] Centanni M, Viceconti N, Luisi S, Reis FM, Gargano L, Maiani F (2002). Reversible increase of serum activin A levels in women with Graves’ disease. J Endocrinol Invest.

[51] Sideras P, Apostolou E, Stavropoulos A, Sountoulidis A, Gavriil A, Apostolidou A (2013). Activin, neutrophils, and inflammation: just coincidence?. Semin Immunopathol.

[52] Sulyok S, Wankell M, Alzheimer C, Werner S (2004). Activin: an important regulator of wound repair, fibrosis, and neuroprotection. Mol Cell Endocrinol.

[53] Hatzis C, Pusztai L, Valero V, Booser DJ, Esserman L, Lluch A (2011). A genomic predictor of response and survival following taxane-anthracycline chemotherapy for invasive breast cancer. JAMA.

[54] Curtis C, Shah SP, Chin SF, Turashvili G, Rueda OM, Dunning MJ (2012). The genomic and transcriptomic architecture of 2,000 breast tumours reveals novel subgroups. Nature.

[55] Kao KJ, Chang KM, Hsu HC, Huang AT (2011). Correlation of microarray-based breast cancer molecular subtypes and clinical outcomes: implications for treatment optimization. BMC Cancer.

[56] Richardson AL, Wang ZC, De Nicolo A, Lu X, Brown M, Miron A (2006). X chromosomal abnormalities in basal-like human breast cancer. Cancer Cell.

[57] Sorlie T, Tibshirani R, Parker J, Hastie T, Marron JS, Nobel A (2003). Repeated observation of breast tumor subtypes in independent gene expression data sets. Proc Natl Acad Sci U S A.

[58] Györffy B, Lanczky A, Eklund AC, Denkert C, Budczies J, Li Q (2010). An online survival analysis tool to rapidly assess the effect of 22,277 genes on breast cancer prognosis using microarray data of 1,809 patients. Breast Cancer Res Treat.

