# Supplementary material for: Follistatin is a metastasis suppressor in a mouse model of HER2-positive breast cancer
Source: Breast Cancer Res. 2017 Jun 5;19:66. doi: 10.1186/s13058-017-0857-y (PMC5460489; doi:10.1186/s13058-017-0857-y)
Supplement: Supplementary file 1 — Supplementary methods. (DOCX 21 kb) [file 13058_2017_857_MOESM1_ESM.docx]

**Supplementary Methods**

*Kaplan-Meier Analyses*. For overall survival in the Curtis dataset ([1](#_ENREF_1)), or metastatic recurrence in the Hatzis ([2](#_ENREF_2)) and Kao ([3](#_ENREF_3)) datasets, the respective patient cohort was stratified into high (upper 10^th^ percentile) and low (remaining 90^th^ percentile) groups. Kaplan-Meier curves were then generated and compared by the log-rank test. *FST, FSTL1, FSTL2, FSTL3 and ESR1* recurrence-free survival analyses were performed using Kaplan-Meier plotter 2014 edition ([4](#_ENREF_4)) computer-generated expression cut-offs as follows: *FST (all breast cancers)*; 1-8465 expression range, computer cut-off=88, low (n=967), high (n=2587). *FST* (Luminal A subtype); 6-26,451 expression range, cut-off=571, low (n=830), high (n=934). *FST* (Luminal B subtype); 21-10,388 expression range, computer cut-off=760, low (n=705), high (n=297). *FST* (HER2 subtype); 29-3059 expression range, computer cut-off=349, low (n=98), high (n=110). *FST* (basal subtype); 36-4533 expression range, computer cut-off=232, low (n=144), high (n=436). *ESR1;* 10-34,801 expression range, computer cut-off=707, low (n=887), high(n=2667)*. FSTL1; 7-20,725 expression range, computer cut-off=3674, low (n=889), high (n=2665). FSTL2;* 5-37,124 expression range, cut-off=6951, low (n=1154), high (n=2400).; *FSTL3*; 9-1710 expression range, cut-off=225, low (n=892), high (n=2662).

**References**

**1. Curtis C, Shah SP, Chin SF, Turashvili G, Rueda OM, Dunning MJ*, et al.* The genomic and transcriptomic architecture of 2,000 breast tumours reveals novel subgroups. Nature 2012;486(7403):346-52.**

**2. Hatzis C, Pusztai L, Valero V, et al. A genomic predictor of response and survival following taxane-anthracycline chemotherapy for invasive breast cancer. JAMA : the journal of the American Medical Association 2011;305(18):1873-81.**

**3. Kao K-J, Chang K-M, Hsu H-C, Huang AT. Correlation of microarray-based breast cancer molecular subtypes and clinical outcomes: implications for treatment optimization. BMC cancer 2011;11:143-.**

**4. Gyorffy B, Lanczky A, Eklund AC, Denkert C, Budczies J, Li Q*, et al.* An online survival analysis tool to rapidly assess the effect of 22,277 genes on breast cancer prognosis using microarray data of 1,809 patients. Breast cancer research and treatment 2010;123(3):725-31.**
