# Supplementary figures and images for: Follistatin is a metastasis suppressor in a mouse model of HER2-positive breast cancer
Source: Breast Cancer Res. 2017 Jun 5;19:66. doi: 10.1186/s13058-017-0857-y (PMC5460489; doi:10.1186/s13058-017-0857-y)

## Slide 1
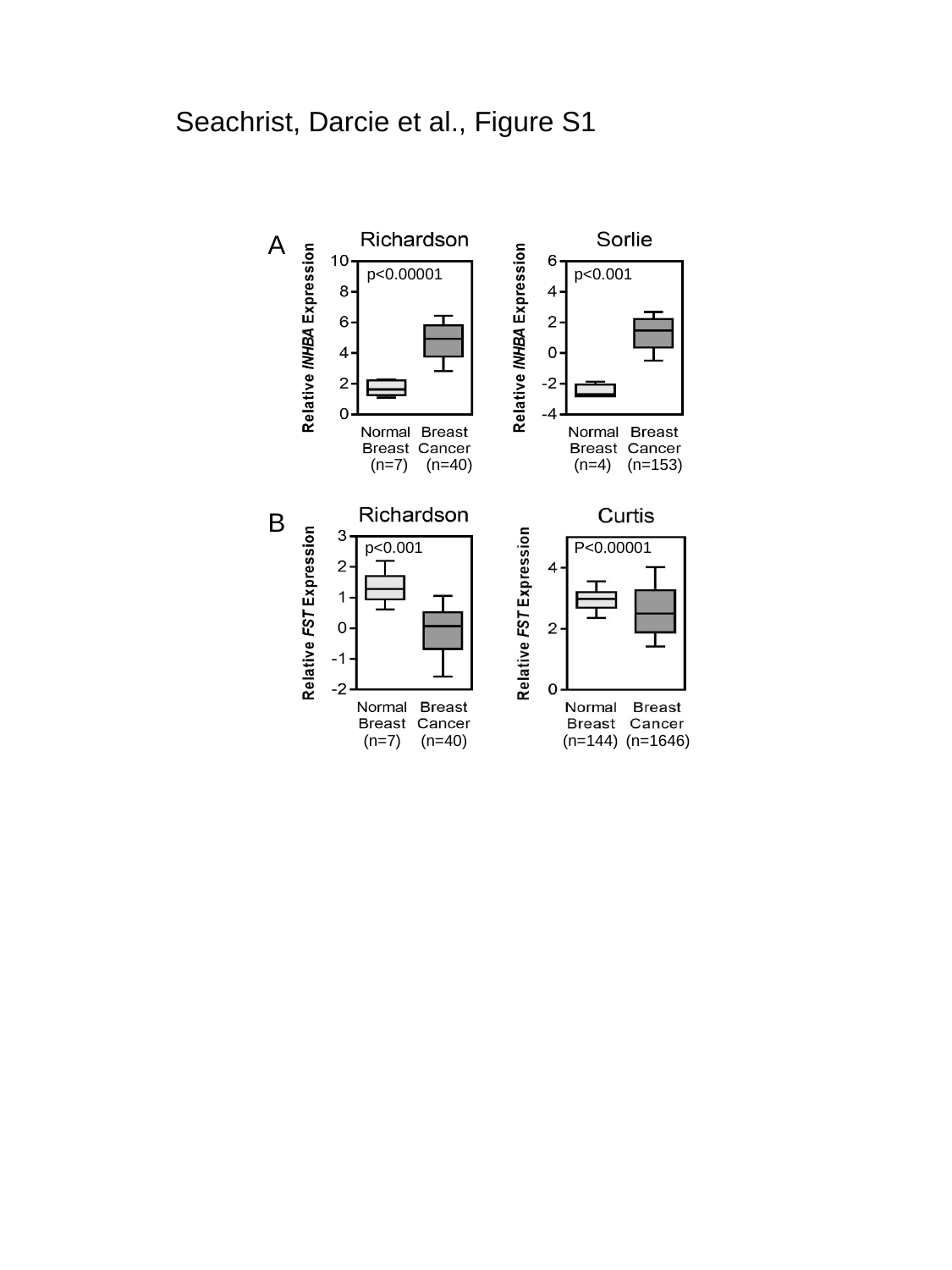

Seachrist, Darcie et al., Figure S1
A
p<0.00001
p<0.001
(n=7)
(n=40)
(n=4)
(n=153)
B
p<0.001
P<0.00001
(n=7)
(n=40)
(n=144)
(n=1646)

Supplement: Supplementary file 2 — Expression of the INHBA/FST axis is altered in human breast cancers. a Relative INHBA expression in normal breast versus breast cancer using publicly available datasets from Richardson et al. [56] and Sorlie et al. [57]. b Relative FST expression in normal breast versus breast cancer as above from Richardson et al. [56] and Curtis et al. [54] datasets. (PPTX 161 kb) [file 13058_2017_857_MOESM2_ESM.pptx]

## Slide 1
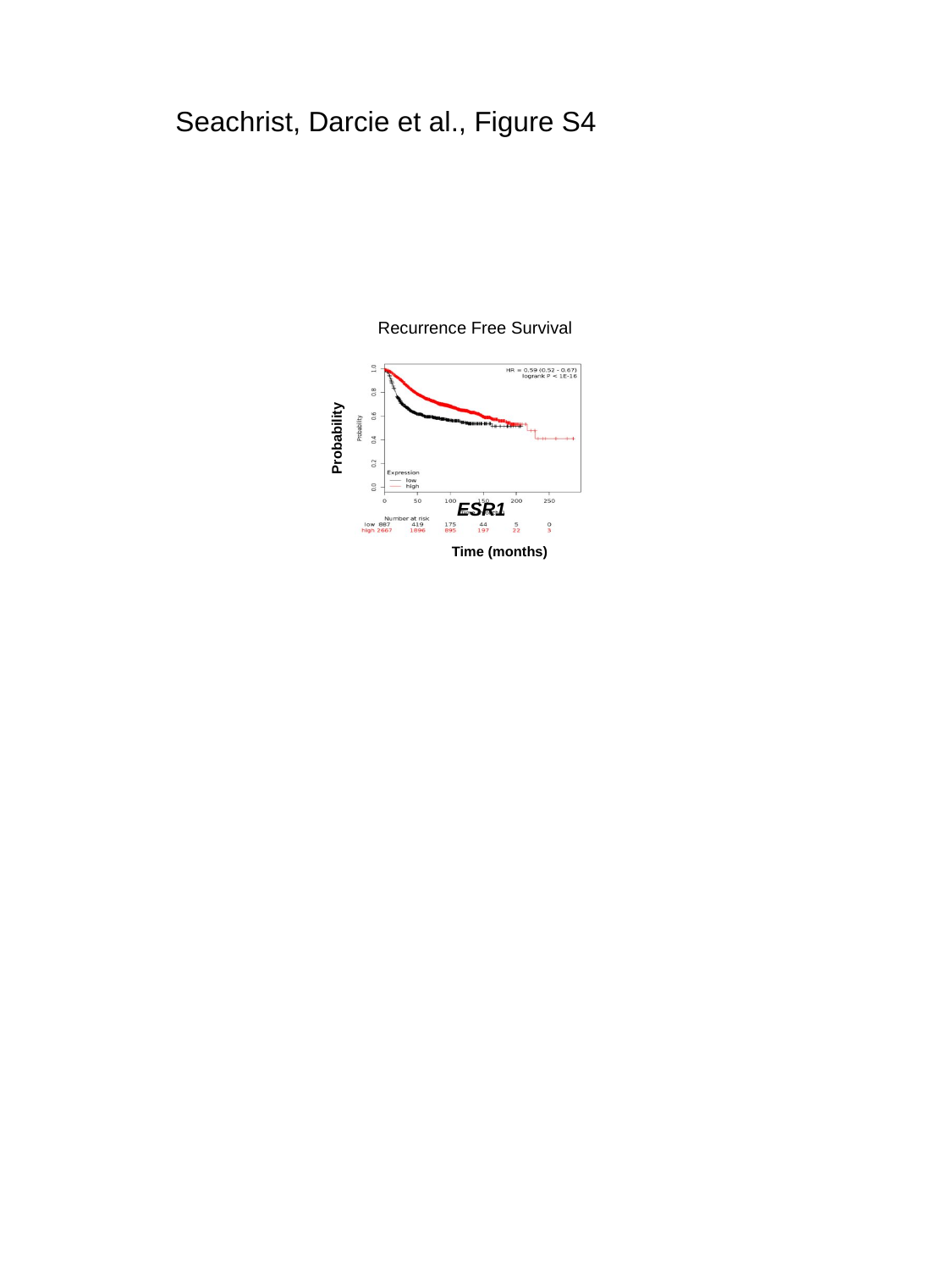

Seachrist, Darcie et al., Figure S4
Recurrence Free Survival
Probability
ESR1
Time (months)

Supplement: Supplementary file 5 — Kaplan-Meier plot demonstrating that ESR1 expression predicts recurrence-free survival similarly to FST in a cohort of over 2800 patients with breast cancer (all subtypes). Patients are stratified in high- and low-expressing groups for ESR1 using optimal cutoffs in the KM Plotter data analysis tool [58]. (PPTX 84 kb) [file 13058_2017_857_MOESM5_ESM.pptx]
