# Supplementary material for: Follistatin is a metastasis suppressor in a mouse model of HER2-positive breast cancer
Source: Breast Cancer Res. 2017 Jun 5;19:66. doi: 10.1186/s13058-017-0857-y (PMC5460489; doi:10.1186/s13058-017-0857-y)
Supplement: Supplementary file 3 — FST expression is reduced in breast cancer cell lines compared with nontransformed mammary epithelial cells. a Western blot analysis of breast cancer cell lines demonstrating loss of FST expression compared with nontransformed (NT) mammary epithelial cells. b FST expression in MCF10A versus MCF10A-Neu stable cell lines that overexpress rat c-Neu/ErbB2 [27]. FST was assessed by quantitative RT-PCR relative to TATA-binding protein (TBP) mRNA (**p < 0.01). (PPTX 1925 kb) [file 13058_2017_857_MOESM3_ESM.pptx]

## Slide 1
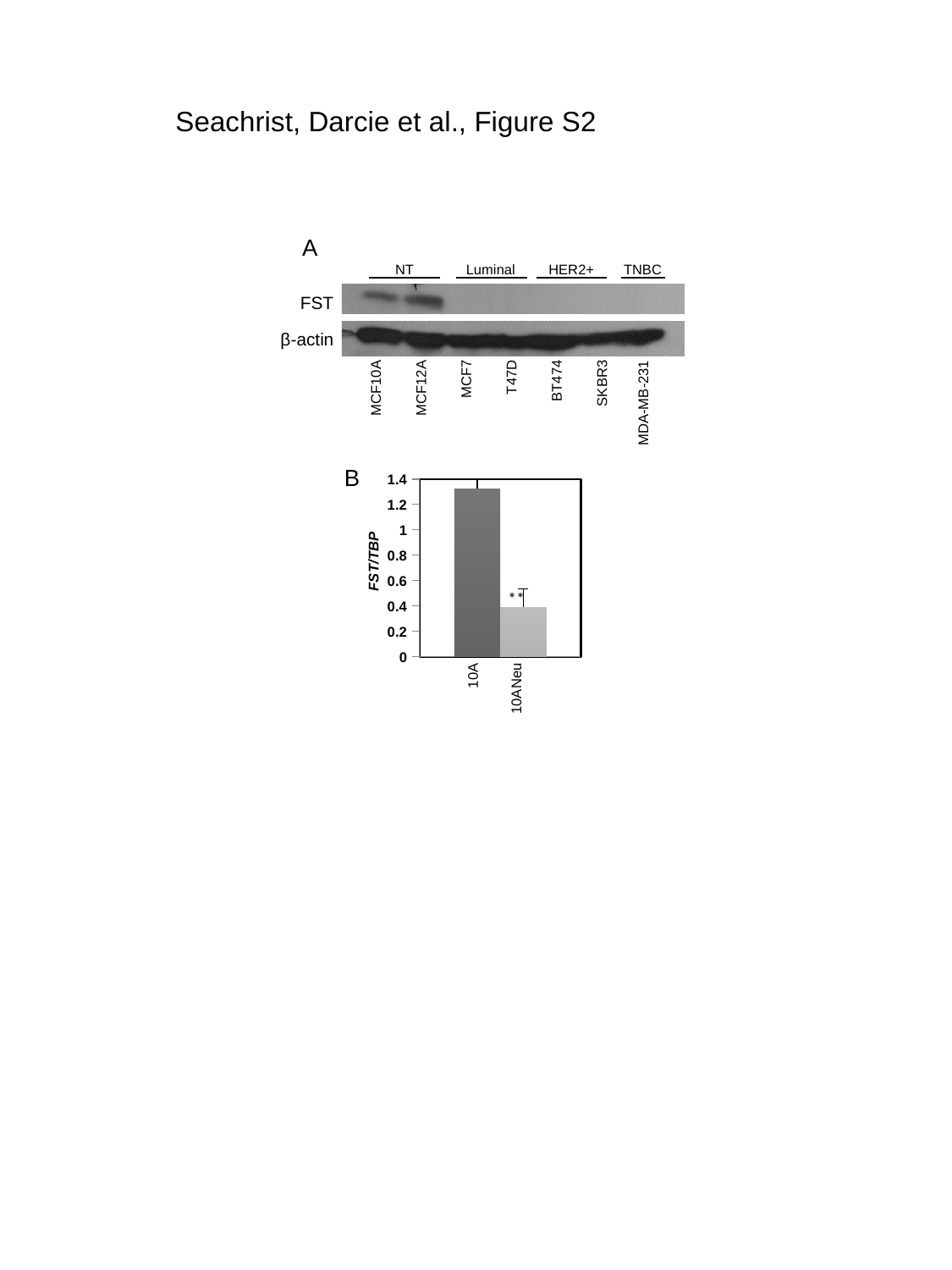

Seachrist, Darcie et al., Figure S2
A
NT
Luminal
HER2+
TNBC
FST
β-actin
T47D
MCF7
BT474
SKBR3
MCF10A
MCF12A
MDA-MB-231
B
### Chart
| Category | 10A | 10ANeu |
|---|---|---|FST/TBP
**
10A
10ANeu
