# Supplementary material for: Follistatin is a metastasis suppressor in a mouse model of HER2-positive breast cancer
Source: Breast Cancer Res. 2017 Jun 5;19:66. doi: 10.1186/s13058-017-0857-y (PMC5460489; doi:10.1186/s13058-017-0857-y)
Supplement: Supplementary file 4 — Follistatin inhibits activin A-induced invasion without impacting proliferation. a MCF10A and 4 T1 cells were treated with vehicle, recombinant human activin A (100 ng/ml), or activin A plus recombinant human FST (400 ng/ml), and cell number was assessed by MTS assay after 72 h. b MCF10A cells that overexpress rat c-Neu (10ANeu) were treated with vehicle, activin A (100 ng/ml), or activin A plus FST (400 ng/ml) for 48 h and plated for invasion assays in modified Boyden chambers + Matrigel overnight with serum as a chemoattractant in addition to follistatin and/or activin A (*p < 0.01 compared with vehicle and † p < 0.01 compared with activin A). (PPTX 115 kb) [file 13058_2017_857_MOESM4_ESM.pptx]

## Slide 1
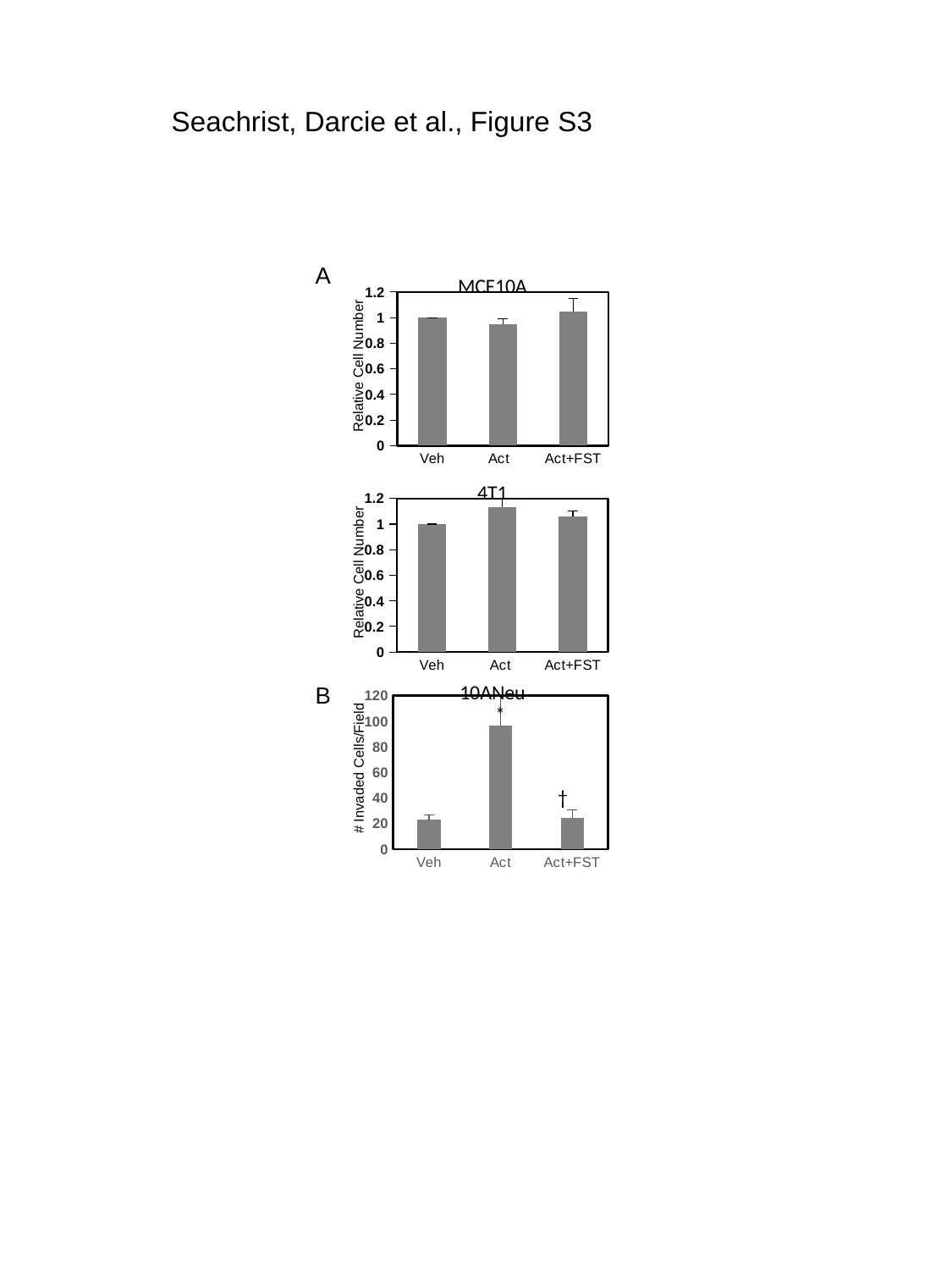

Seachrist, Darcie et al., Figure S3
A
MCF10A
### Chart
| Category | |
|---|---|
| Veh | 1.0 |
| Act | 0.9502992948936758 |
| Act+FST | 1.0462129699874056 |Relative Cell Number
4T1
### Chart
| Category | |
|---|---|
| Veh | 1.0 |
| Act | 1.132025172152648 |
| Act+FST | 1.05674736410005 |Relative Cell Number
10ANeu
B
### Chart
| Category | |
|---|---|
| Veh | 22.5 |
| Act | 96.48333333333333 |
| Act+FST | 24.266666666666666 |*
# Invaded Cells/Field
†
