# Supplementary material for: Follistatin is a metastasis suppressor in a mouse model of HER2-positive breast cancer
Source: Breast Cancer Res. 2017 Jun 5;19:66. doi: 10.1186/s13058-017-0857-y (PMC5460489; doi:10.1186/s13058-017-0857-y)
Supplement: Supplementary file 6 — FST expression is associated with overall survival and metastasis in multiple cohorts of patients with breast cancer. Tumors are stratified into the highest 10% and lowest (remaining 90%) FST-expressing groups for each dataset as follows: Curtis et al. [54] reported FST low (n = 1774), FST high (n = 197); Hatzis et al. [53] reported FST low (n = 457), FST high (n = 51); and Kao et al. [55] reported FST low (n = 294), FST high (n = 33). FST expression does not predict recurrence to bone in the Bos et al. cohort [24]: FST low (n = 149), FST high (n = 17). (PPTX 109 kb) [file 13058_2017_857_MOESM6_ESM.pptx]

## Slide 1
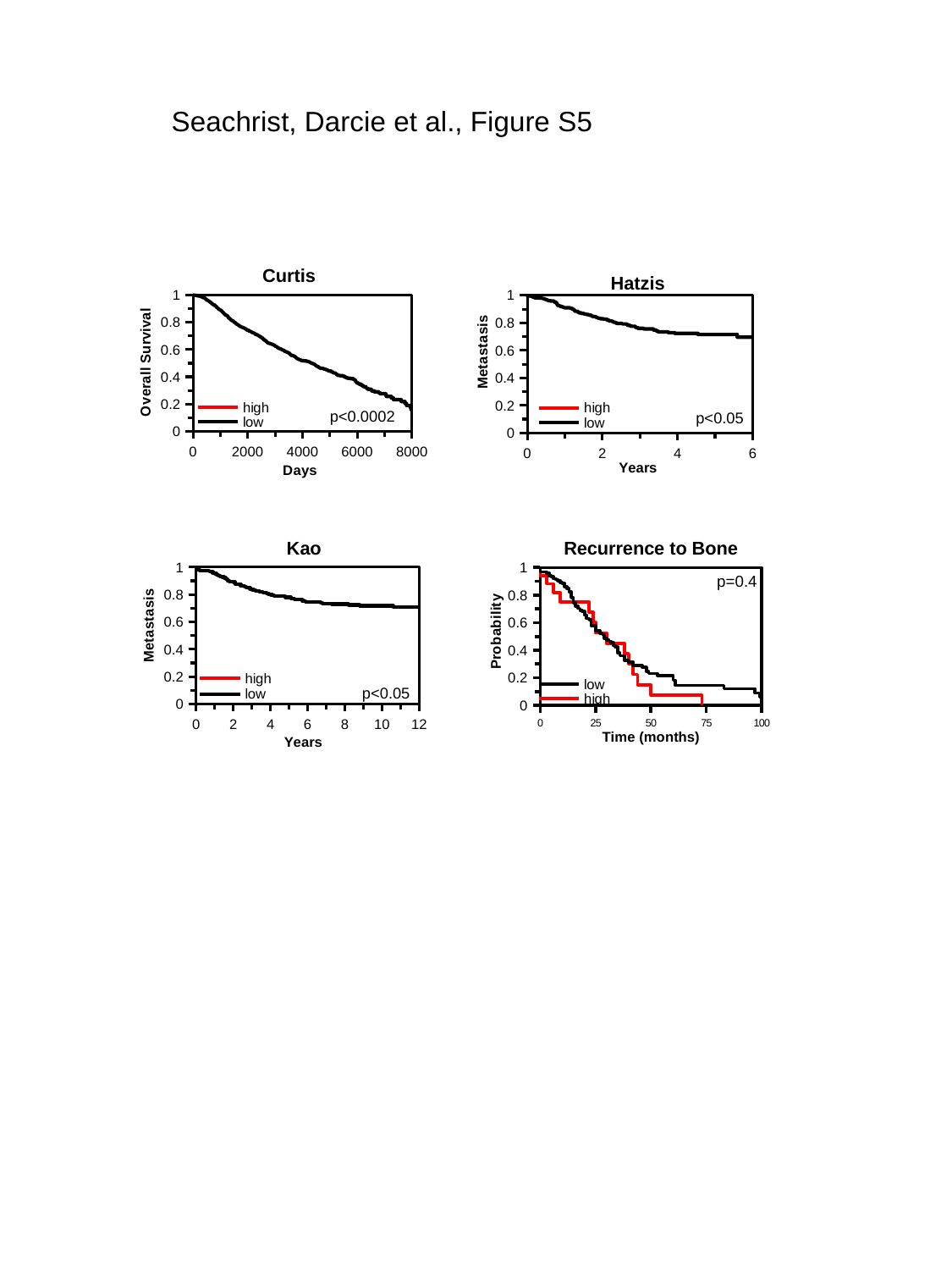

Seachrist, Darcie et al., Figure S5
### Chart
| Category | | |
|---|---|---|Curtis
p<0.0002
### Chart
| Category | | |
|---|---|---|Hatzis
p<0.05
### Chart
| Category | | |
|---|---|---|Kao
p<0.05
### Chart
| Category | | |
|---|---|---|Recurrence to Bone
p=0.4
Time (months)
