# Supplementary material for: Follistatin is a metastasis suppressor in a mouse model of HER2-positive breast cancer
Source: Breast Cancer Res. 2017 Jun 5;19:66. doi: 10.1186/s13058-017-0857-y (PMC5460489; doi:10.1186/s13058-017-0857-y)
Supplement: Supplementary file 7 — FST expression predicts recurrence-free survival independent of breast cancer subtype. Kaplan-Meier plots demonstrating the association of high FST expression with recurrence-free survival of luminal A, luminal B, HER2, and basal subtypes of breast cancer. High and low FST-expressing groups are stratified using optimal cutoffs in the KM Plotter data analysis tool [58]. (PPTX 330 kb) [file 13058_2017_857_MOESM7_ESM.pptx]

## Slide 1
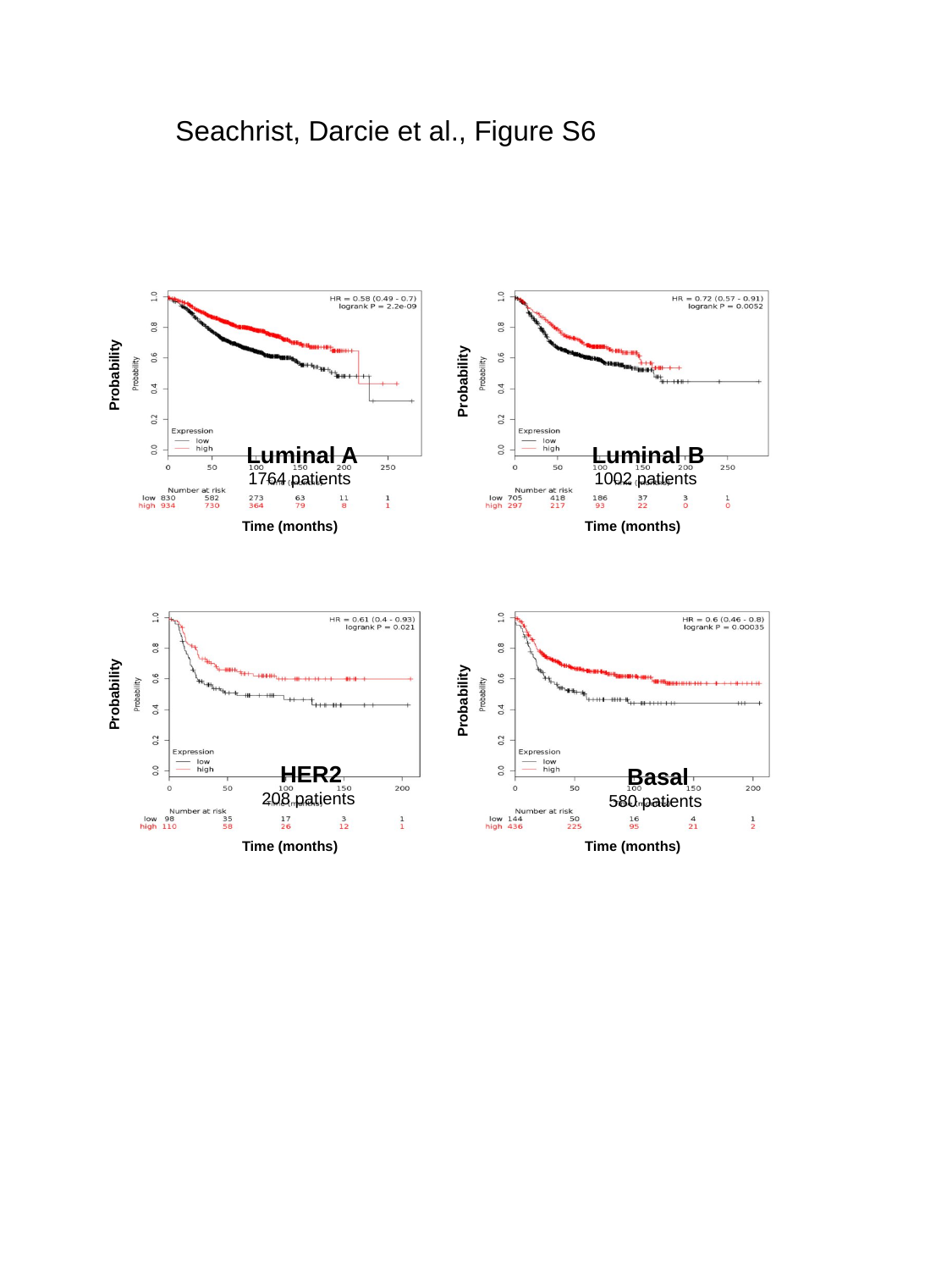

Seachrist, Darcie et al., Figure S6
Probability
Luminal A
1764 patients
Time (months)
Probability
HER2
208 patients
Time (months)
Probability
Luminal B
1002 patients
Time (months)
Probability
Basal
580 patients
Time (months)
