# Supplementary material for: Follistatin is a metastasis suppressor in a mouse model of HER2-positive breast cancer
Source: Breast Cancer Res. 2017 Jun 5;19:66. doi: 10.1186/s13058-017-0857-y (PMC5460489; doi:10.1186/s13058-017-0857-y)
Supplement: Supplementary file 8 — Kaplan-Meier plots demonstrating that FSTL1, FSTL2, and FSTL3 expression also predict recurrence-free survival in a cohort of over 2800 patients with breast cancer. Patients are stratified in high- and low-expressing groups for each gene using optimal cutoffs in the KM Plotter data analysis tool [58]. (PPTX 185 kb) [file 13058_2017_857_MOESM8_ESM.pptx]

## Slide 1
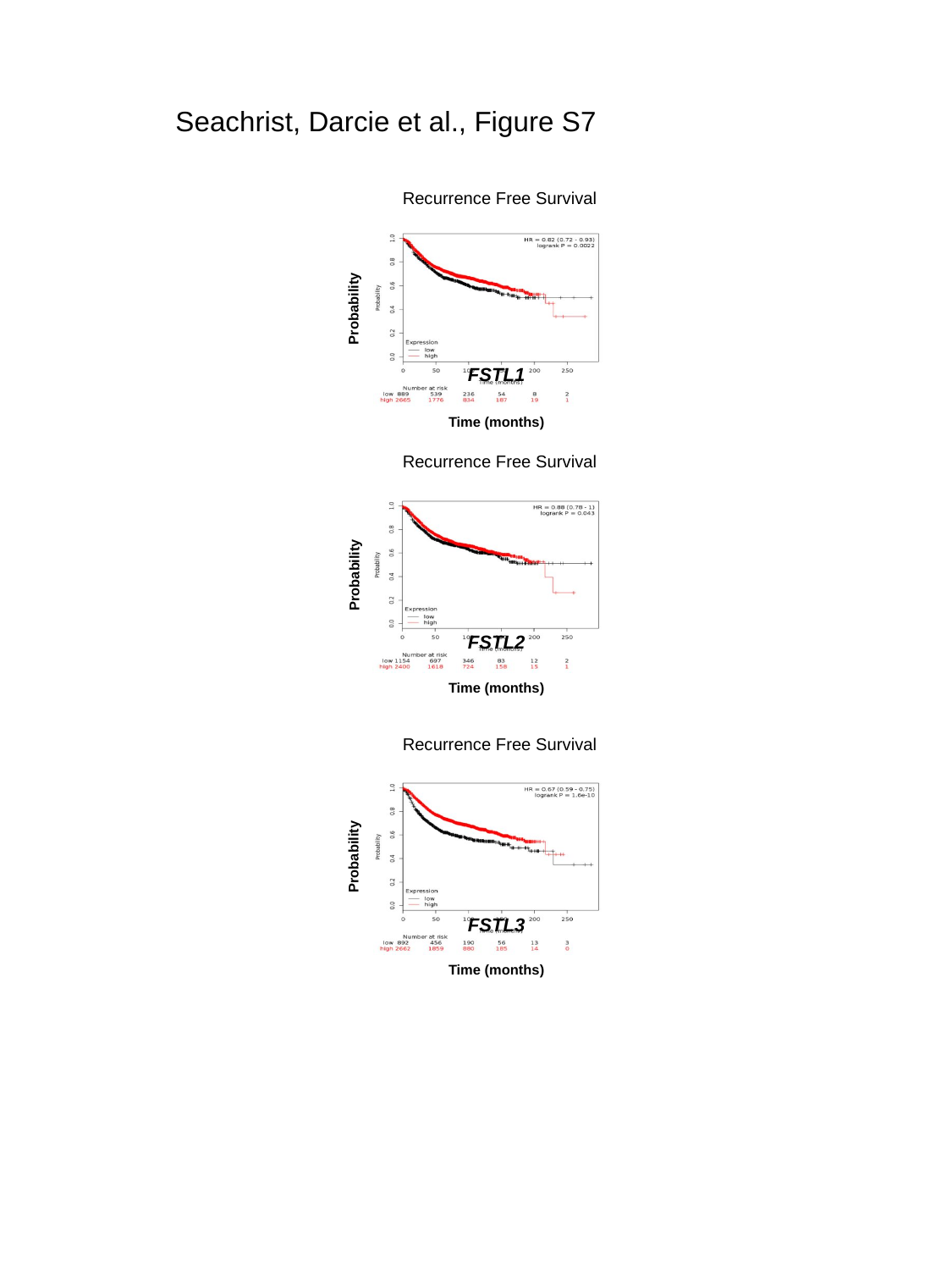

Seachrist, Darcie et al., Figure S7
Recurrence Free Survival
Probability
FSTL1
Time (months)
Recurrence Free Survival
Probability
FSTL2
Time (months)
Recurrence Free Survival
Probability
FSTL3
Time (months)
