# Supplementary material for: Follistatin is a metastasis suppressor in a mouse model of HER2-positive breast cancer
Source: Breast Cancer Res. 2017 Jun 5;19:66. doi: 10.1186/s13058-017-0857-y (PMC5460489; doi:10.1186/s13058-017-0857-y)
Supplement: Supplementary file 9 — FST overexpression in mouse mammary epithelia. a Follistatin-like 3 (Fstl3) is downregulated in HER 2/Neu-induced mouse mammary tumors compared with normal mammary glands. Fstl3 expression in mammary glands and HER 2/Neu tumors was determined using data from a published microarray study of these tumors (*p < 0.001) [21]. b and c Fst is overexpressed in mammary epithelia of founder female mice and Fst-overexpressing founder offspring (in mixed FVB/N background) as determined by quantitative RT-PCR. d Fst expression in uterus, lung, and liver of 10- to 12-month-old MMTV-Fst female single transgenic mice (n = 3) and wild-type littermates (n = 2). e Representative whole mounts of adult mammary glands from female Fst-overexpressing founder offspring and littermate wild-type mice in the C57BL/6:SJ:FVB background. f Schematic of MMTV-Fst transgene and breeding paradigm used to obtain FST/Neu bitransgenic mice with restored FST expression. (PPTX 712 kb) [file 13058_2017_857_MOESM9_ESM.pptx]

## Slide 1
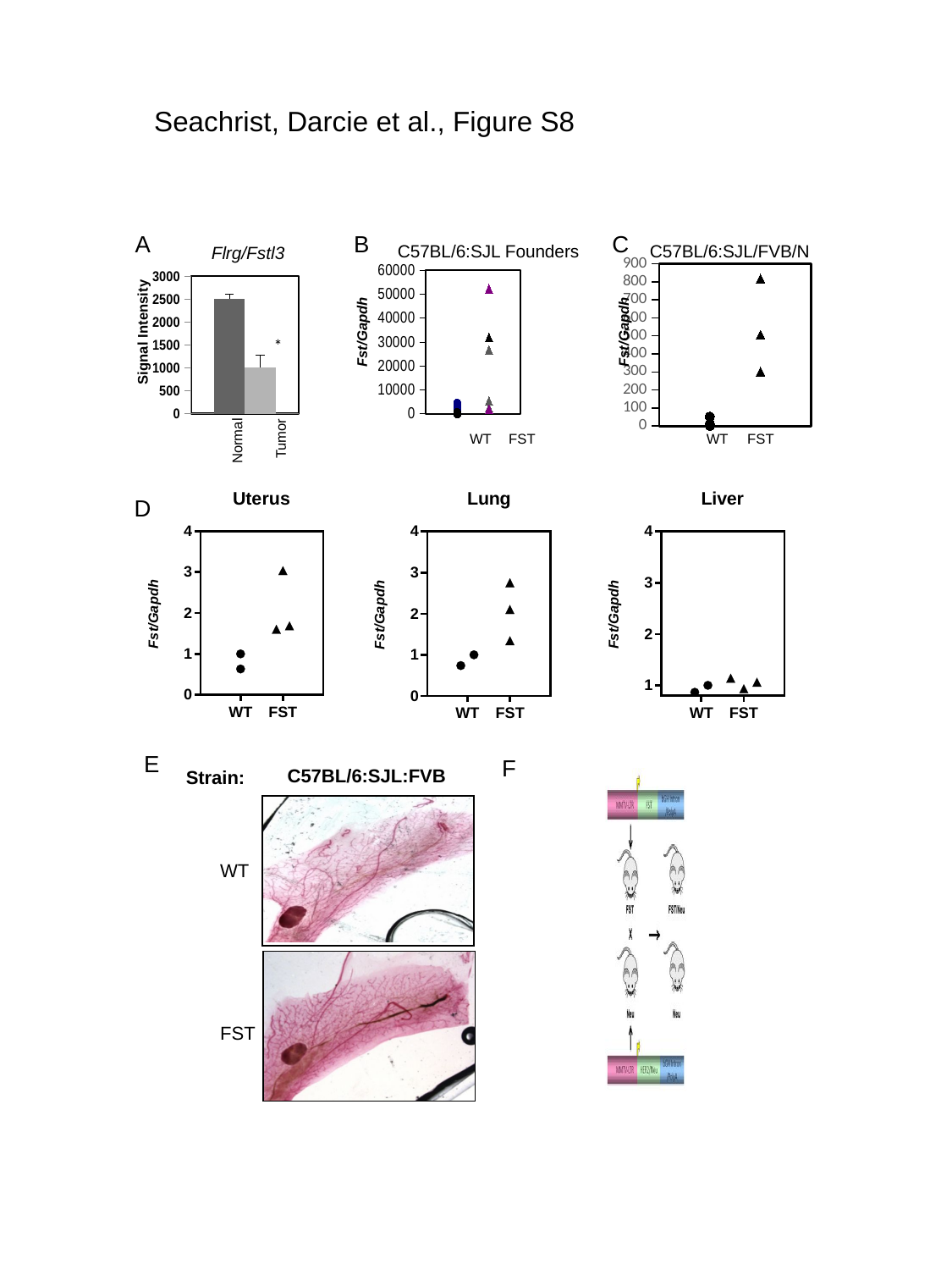

Seachrist, Darcie et al., Figure S8
A
B
C
C57BL/6:SJL Founders
C57BL/6:SJL/FVB/N
Flrg/Fstl3
### Chart
| Category | | | | | | | | | | | | | | | | | |
|---|---|---|---|---|---|---|---|---|---|---|---|---|---|---|---|---|---|
### Chart
| Category | | | | | | | | | | | | | | | | | | | | | | | | | | | | | |
|---|---|---|---|---|---|---|---|---|---|---|---|---|---|---|---|---|---|---|---|---|---|---|---|---|---|---|---|---|---|
### Chart
| Category | Normal | Tumor |
|---|---|---|Fst/Gapdh
Fst/Gapdh
Signal Intensity
*
Tumor
WT
FST
WT
FST
Normal
D
E
F
C57BL/6:SJL:FVB
Strain:
WT
FST
